# JC Virus infected choroid plexus epithelial cells produce extracellular vesicles that infect glial cells independently of the virus attachment receptor

**DOI:** 10.1371/journal.ppat.1008371

**Published:** 2020-03-04

**Authors:** Bethany A. O’Hara, Jenna Morris-Love, Gretchen V. Gee, Sheila A. Haley, Walter J. Atwood

**Affiliations:** 1 Department of Molecular Biology, Cell Biology, and Biochemistry, Brown University, Providence, Rhode Island, United States of America; 2 Graduate Program in Pathobiology, Brown University, Providence, Rhode Island, United States of America; 3 MassBiologics, University of Massachusetts Medical School, Worcester, Massachusetts, United States of America; Wake Forest University, UNITED STATES

## Abstract

The human polyomavirus, JCPyV, is the causative agent of progressive multifocal leukoencephalopathy (PML) in immunosuppressed and immunomodulated patients. Initial infection with JCPyV is common and the virus establishes a long-term persistent infection in the urogenital system of 50–70% of the human population worldwide. A major gap in the field is that we do not know how the virus traffics from the periphery to the brain to cause disease. Our recent discovery that human choroid plexus epithelial cells are fully susceptible to virus infection together with reports of JCPyV infection of choroid plexus in vivo has led us to hypothesize that the choroid plexus plays a fundamental role in this process. The choroid plexus is known to relay information between the blood and the brain by the release of extracellular vesicles. This is particularly important because human macroglia (oligodendrocytes and astrocytes), the major targets of virus infection in the central nervous system (CNS), do not express the known attachment receptors for the virus and do not bind virus in human tissue sections. In this report we show that JCPyV infected choroid plexus epithelial cells produce extracellular vesicles that contain JCPyV and readily transmit the infection to human glial cells. Transmission of the virus by extracellular vesicles is independent of the known virus attachment receptors and is not neutralized by antisera directed at the virus. We also show that extracellular vesicles containing virus are taken into target glial cells by both clathrin dependent endocytosis and macropinocytosis. Our data support the hypothesis that the choroid plexus plays a fundamental role in the dissemination of virus to brain parenchyma.

## Author summary

JC polyomavirus (JCPyV) is a common human pathogen that causes a central nervous system demyelinating disease known as progressive multifocal leukoencephalopathy (PML). To cause PML, JCPyV has to traffic from peripheral tissues to the central nervous system (CNS) and invade glial cells. In previous work we found that choroid plexus epithelial cells express receptors for JCPyV in vivo and are fully susceptible to virus infection in vitro. In contrast, glial cells do not express the receptors for JCPyV and virus does not bind to these cells in human tissue sections. Because choroid plexus epithelial cells are known to relay information between the blood and the brain using extracellular vesicles we hypothesized that this could be important for JCPyV neuroinvasion. We found that JCPyV infected choroid plexus epithelial cells produce extracellular vesicles containing JCPyV virions and that these extracellular vesicles transmit the infection to human glial cells independently of the virus attachment receptor. These findings support our hypothesis that the choroid plexus is important in the dissemination of virus to the brain to initiate disease.

## Introduction

JCPyV, a human polyomavirus, establishes a lifelong persistent infection in over half the world’s population [1]. In immunosuppressed or immunomodulated patients JCPyV spreads to the central nervous system where infection of glial cells leads to a rapidly progressing and severely debilitating demyelinating disease known as progressive multifocal leukoencephalopathy or PML [1–5]. PML is a significant complication in AIDS patients and is considered an AIDS defining illness despite advances in anti-retroviral therapy [2]. In the last decade PML has also emerged as a fatal complication in multiple sclerosis patients being treated with powerful immunomodulators [6–11]. The mechanisms that contribute to the reactivation and spread of this relatively benign virus from the periphery to the CNS to cause debilitating and often fatal disease are not understood.

Cell culture models using purified JCPyV virions have shown that the virus requires the sialic acid-containing attachment receptor, lactoseries tetrasaccharide C (LSTc), to bind target cells [12–15]. The virus then uses one of at least three isoforms of serotonin receptor to enter the cells by clathrin dependent mechanisms [13, 14, 16]. In human tissue sections both the attachment receptor and the entry receptors are expressed on human kidney tubule epithelial cells and on choroid plexus epithelial cells and virus binds to these cells in a sialic acid dependent manner [17]. In contrast, neither oligodendrocytes nor astrocytes, the major targets of virus infection in the CNS, express the attachment receptor and neither bind virus [17]. This is consistent with the accumulation of mutations in the sialic acid binding pocket of the virus found in the CSF and brain parenchyma of patients with PML [18–20]. This observation suggests that there is no selective pressure to maintain the sialic acid binding phenotype once virus has invaded brain parenchyma.

The choroid plexus is covered by a layer of specialized epithelial cells (CPE) that are bound by tight junctions, and form the blood-cerebrospinal fluid barrier (BCSFB). The BCSFB separates the cerebrospinal fluid (CSF) from the peripheral blood and restricts the migration of both cells and microbes between the periphery, CSF, and brain [21]. The choroid plexus is also known to respond to inflammatory signals by relaying information between the blood and the brain using extracellular vesicles [22–25]. Extracellular vesicles derived from choroid plexus epithelial cells are capable of delivering specific cargo to distant sites within the CNS [26].

Because choroid plexus epithelial cells (CPE) are susceptible to virus infection both in vivo and in vitro and because they are known to communicate between blood and brain using extracellular vesicles (EV), we asked whether they might be responsible for transmitting JCPyV to human glia in EV independently of the known sialic acid attachment receptor LSTc [22, 26–30]. To study the role of choroid plexus derived extracellular vesicles in dissemination of JCPyV to the CNS in depth we immortalized primary human choroid plexus epithelial cells with human telomerase (hTert) and showed that they maintain the properties of the primary cells. Following virus infection, both the primary CPE cells (CPE-Primary) and the immortalized CPE cell line (CPE-Line) produced virus containing extracellular vesicles that readily transmitted the infection to human glial cells independently of the virus attachment receptor. EV mediated infection could not be neutralized by anti-viral antisera indicating that this mechanism of viral spread may allow the virus to escape immune recognition. We also studied the mechanism of EV entry into target cells and found that extracellular vesicle uptake was dependent on both macropinocytosis and clathrin dependent endocytosis. These data suggest that the choroid plexus is likely a major player in the infectious spread of virus into brain parenchyma.

## Results

### Establishment of an immortalized line of CPE

**Fig 1:**
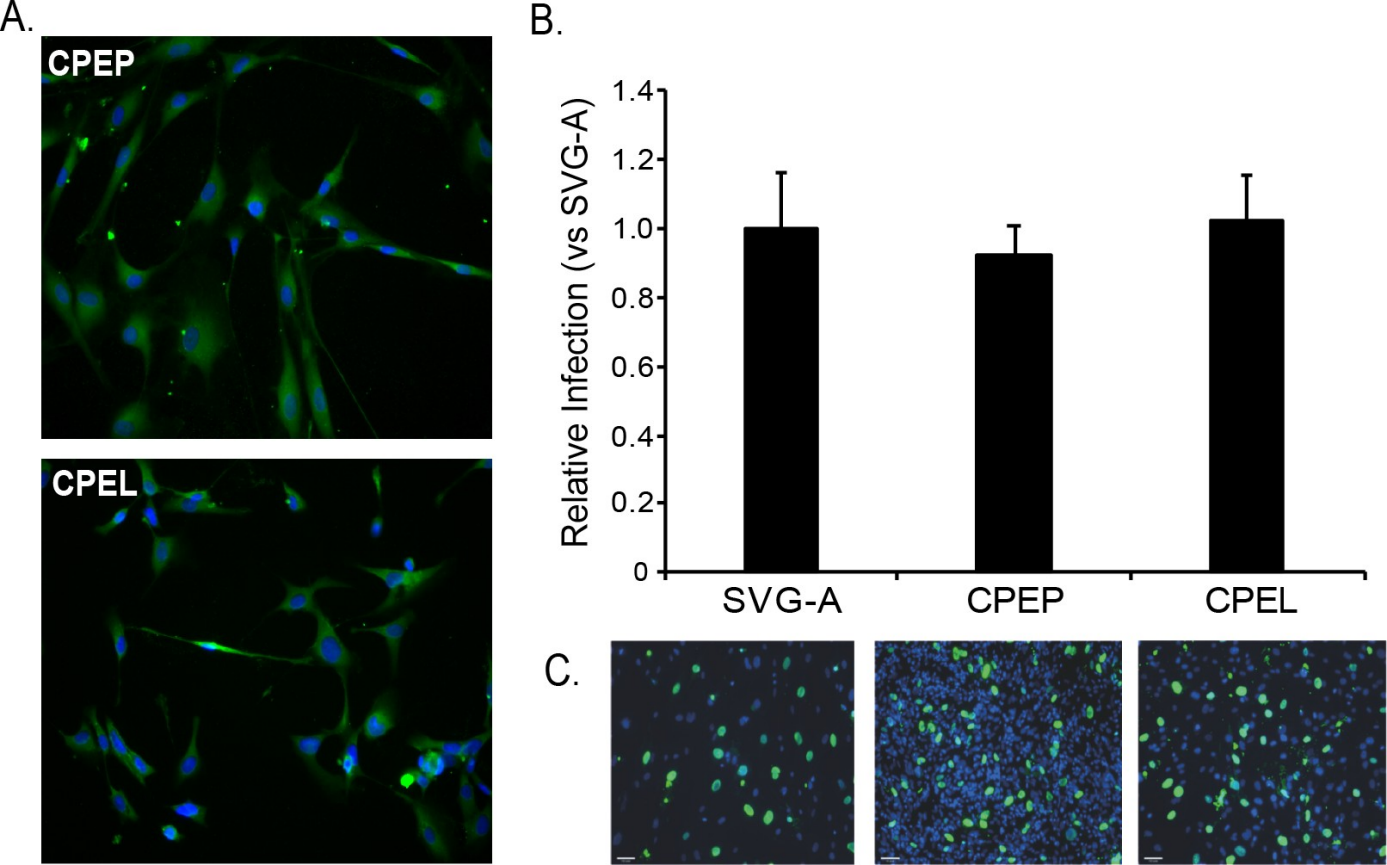
hTert immortalized human choroid plexus epithelial cells support JCPyV infection. (A) Choroid plexus epithelial cells immortalized by serial infection with hTert lentivirus are morphologically similar to primary choroid plexus epithelial cells and express transthyretin (green; DAPI in blue). (B) Quantification of virus infection in primary and immortalized CPE cells compared to infection of the human glial cell line, SVG-A based on expression of the late viral protein VP1. VP1 expression was normalized relative to the infection in SVG-A control cells and set to 1. (C) Indirect immunofluorescent analysis of VP1 positive cells in infected cultures at 5 days post infection (VP1, green; DAPI, blue).

Primary human choroid plexus epithelial cells were serially transduced and immortalized with a lentiviral construct expressing hTert. The CPE cell line (CPEL) was characterized using short tandem repeat analysis (STR) and found to be identical to the parental primary CPE cells (CPEP) **(S1 Table)**. The cells also expressed the CPE marker transthyretin and are as susceptible to infection with purified JCPyV virions as the parental primary cells and the human T-antigen transformed glial cell line, SVG-A **(Fig 1A, 1B and 1C)**.

### The primary CPE cells and the CPE cell line produce similar amounts of extracellular vesicles

**Fig 2:**
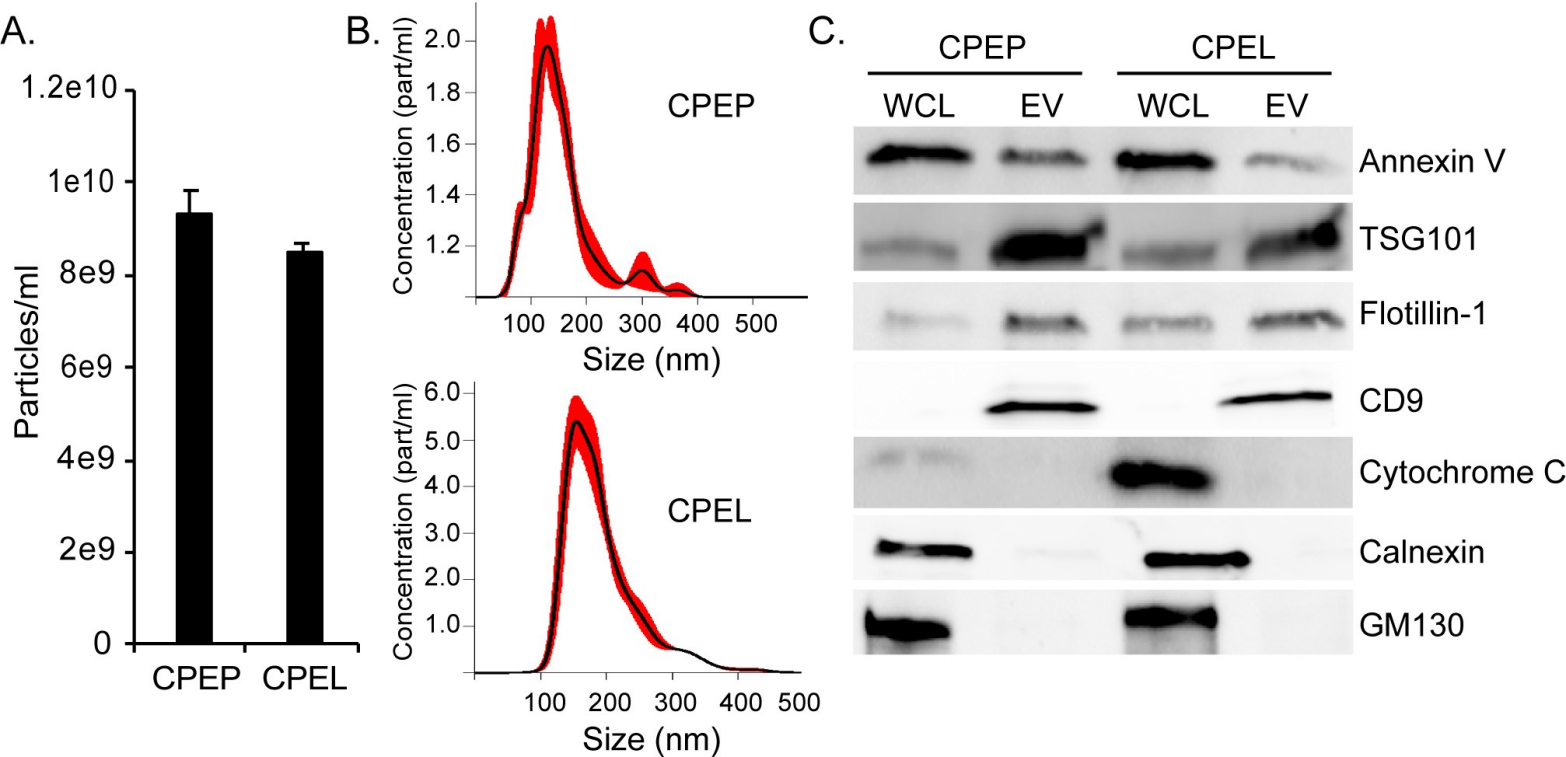
Extracellular vesicles concentrated from CPEP and CPEL cells display similar characteristics and express extracellular vesicle markers. (A) Nanoparticle tracking analysis of extracellular vesicles (EV) produced by primary and immortalized choroid plexus epithelial cells. (B) EV size and distribution is similar between CPEP and CPEL cells. (C) Extracellular vesicles concentrated from CPEP and CPEL cells by differential centrifugation express the characteristic EV markers Annexin V, TSG101, Flotillin-1, and CD9. Markers of contaminating cellular organelles (cytochrome c, calnexin, GM130) were detected in whole cell lysates but not in EV fractions.

Extracellular vesicles were isolated from the primary CPE (CPEP) and from the CPE cell line (CPEL) by differential ultracentrifugation and characterized by nanoparticle tracking analysis (NTA) for particle size and concentration and by Western blot for EV-associated markers. NTA shows that both cell types produce EV that have a size distribution consistent with their characterization as small extracellular vesicles **(Fig 2A and 2B)**. EV isolated from the primary cells and from the cell line also expressed characteristic markers including Annexin V, TSG101, Flotillin-1, and CD9 and did not express markers of contaminating cellular organelles including cytochrome c, calnexin, and GM130 **(Fig 2C)**. These proteins were expressed in whole cell lysates (WCL) from both cell types **(Fig 2C)**.

### Extracellular vesicles isolated from JCPyV infected CPEP and CPEL cells contain virus

**Fig 3:**
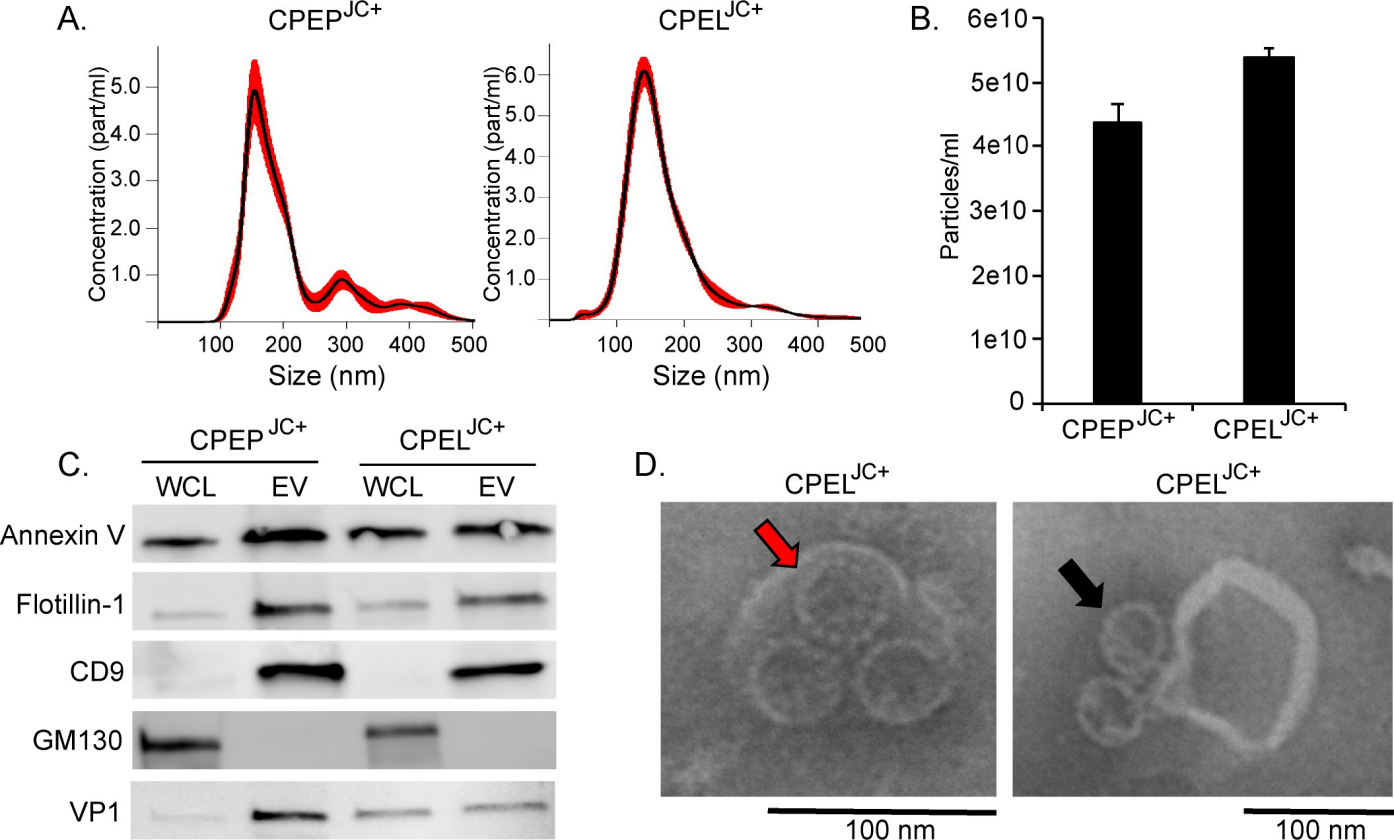
JCPyV associates with vesicles from infected CPEP and CPEL cells. (A) Nanoparticle tracking analysis of extracellular vesicles produced by JCPyV infected primary and immortalized CPE cells show similar vesicle size and distribution, averaging 150-200nm (B) EV yield from infected CPE cultures as quantified by NTA. (C) EV from infected CPEP and CPEL cells express characteristic EV markers and the major viral capsid protein VP1 is found in the EV fraction. (D) Transmission electron microscopy of EV from infected CPE cells shows viral particles bound to the outside of EV and enclosed within EV.

EV isolated from virus-infected CPEP and CPEL cells were quantitatively and qualitatively similar to the EV produced by uninfected cells **(Fig 3A and 3B)**. EVs from both CPEP cells and CPEL cells were positive for the EV associated markers annexin V, Flotillin-1, and CD9 and negative for GM130 **(Fig 3C)**. EV from both the primary cells and the cell line were positive for the major viral capsid protein, VP1, indicating that the virus associated with the extracellular vesicles **(Fig 3C)**. Virus particles were then found both within EV **(Fig 3D-red arrow)** and attached to the outside of some EV **(Fig 3D-black arrow)** by transmission electron microscopy. The size of the extracellular vesicles was consistent with the nanoparticle tracking analysis (NTA) data and the size of the virions approximate 45nm, the known size of JCPyV particles.

### Extracellular vesicles isolated from both the primary CPE cells and from the CPE cell line readily transmitted the infection to permissive human glial cells but not to non-permissive cells

**Fig 4:**
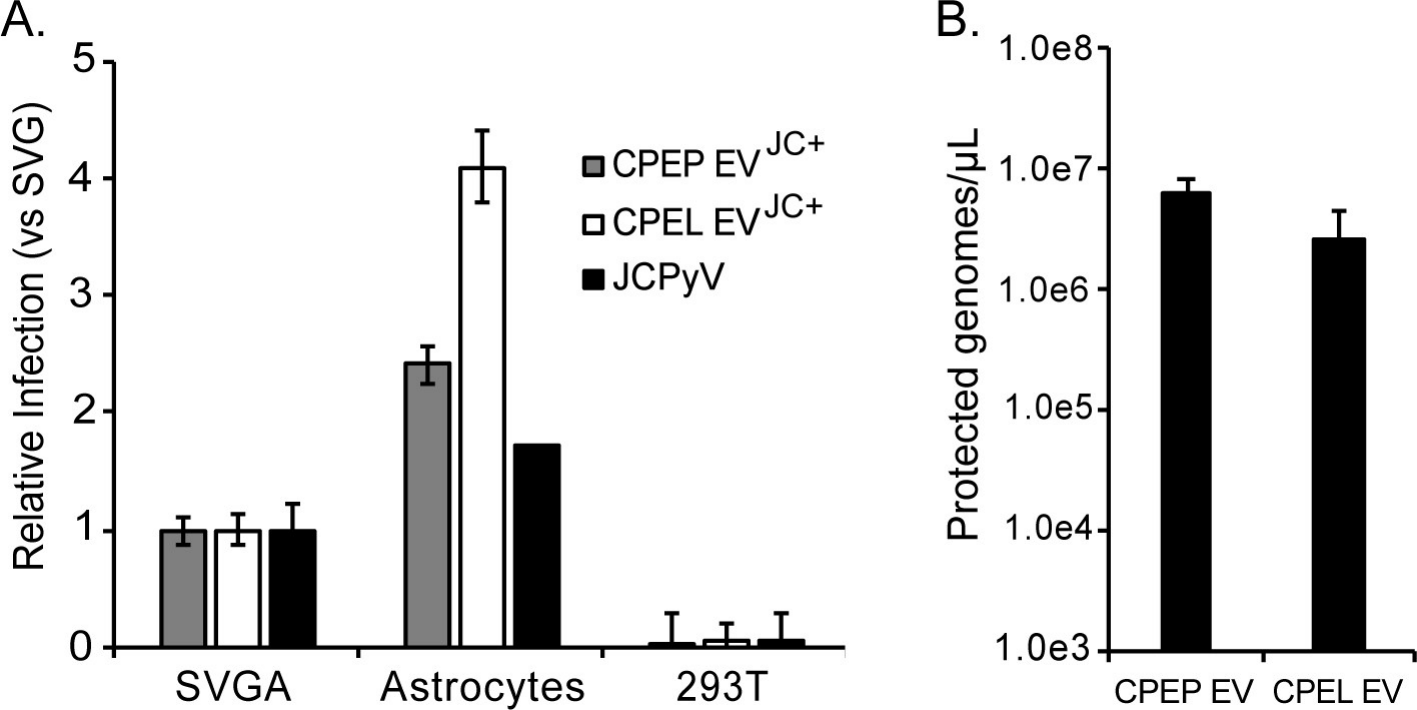
Extracellular vesicles from JCPyV infected CPEP and CPEL cells readily transmit the infection to SVG-A cells and to primary human astrocytes but not to HEK293T cells. (A) CPEP EV^JC+^, CPEL EV^JC+^, and purified JCPyV were used to infect SVG-A, primary human astrocyte, and HEK293T cells. VP1 was quantified by indirect immunofluorescent analysis at 4 days post infection. Infection is normalized relative to the infection in SVG-A control cells which was set to 1. (B) Quantification of protected viral genomes in CPE origin EV^JC+^. Infectious EV preparations were analyzed by qPCR for total protected viral genome content.

Extracellular vesicles isolated from infected primary CPE cells and from the CPE cell line were purified by differential ultracentrifugation and used to challenge SVG-A, primary human astrocytes, and HEK293T cells. Extracellular vesicles from infected CPEP and CPEL cells readily transmitted the infection to SVG-A cells and to primary astrocytes but not to non-permissive HEK293T cells **(Fig 4A)**. The level of infection in SVG-A was set to 1 to compare the efficiency of transmission of the CPEP and CPEL derived EV to SVG-A and primary astrocytes. Infectious EV derived from both the CPEP and CPEL cells were significantly more efficient at transmitting the infection to primary astrocytes than to SVG-A cells **(Fig 4A)**. The amount of viral genomes associated with EV isolated from CPEP and CPEL cells was quantified by qPCR on DNAse treated EV and found to be equivalent **(Fig 4B).**

### EV-mediated infection of glial cells is receptor independent

**Fig 5:**
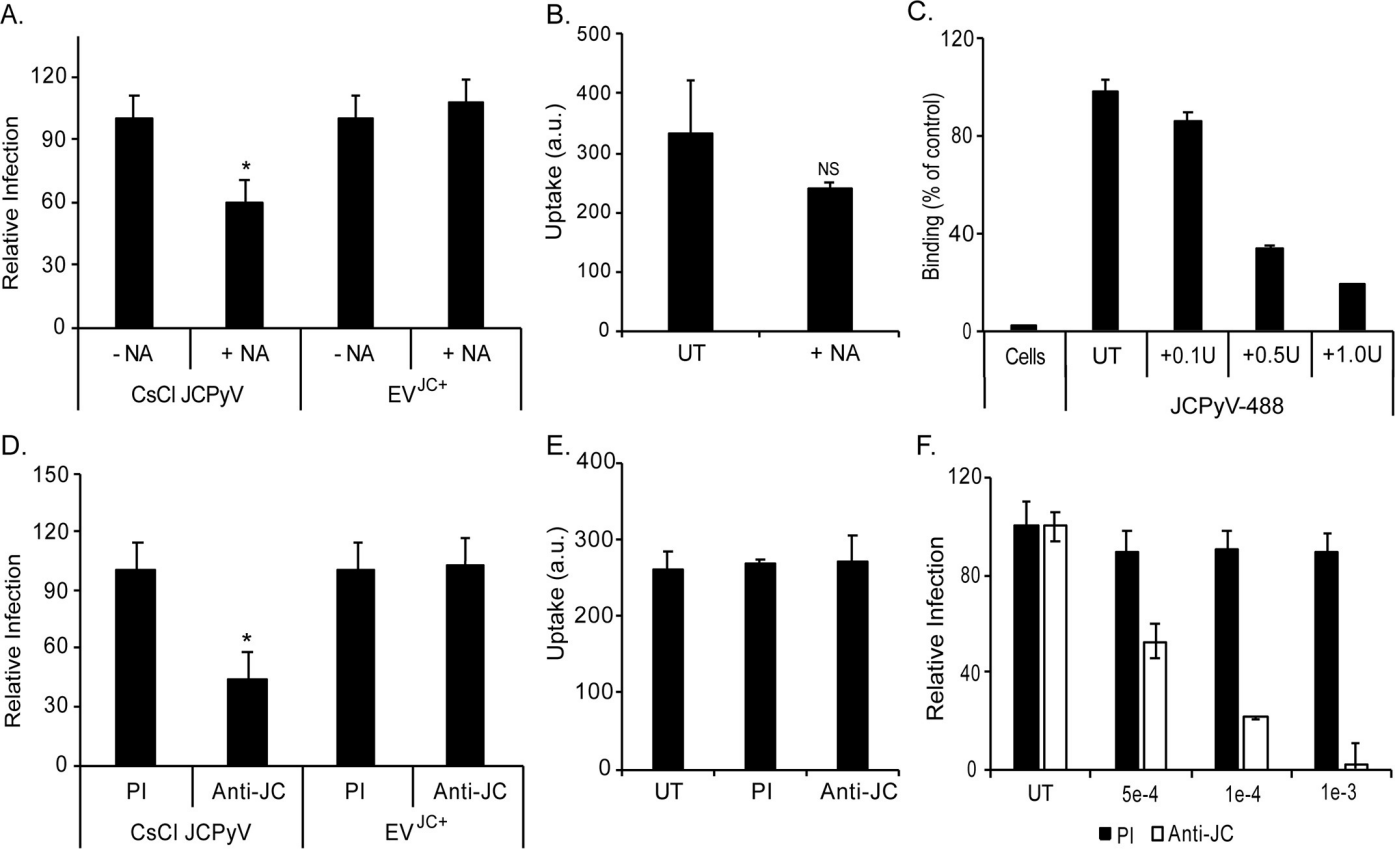
EV-mediated infection of glial cells is not sensitive to treatment with neuraminidase and is not blocked by neutralizing antibodies. (A) Neuraminidase treatment (+NA) of SVG-A cells reduces infection by purified virus (CsCl JCPyV) but has no effect on EV mediated infection (EV^JC+^). (B) Uptake of PKH67 labeled vesicles is not significantly reduced on neuraminidase (+NA) treated SVG-A cells compared to untreated cells (UT). (C) Titration of NA treatment on SVGA cells showed a dose dependent decrease in purified JCPyV-488 binding as NA concentration is increased. (D) Pretreatment with anti-JC blocking antibody inhibits CsCl JCPyV infection but has no effect on EV^JC+^ mediated infection in SVG-A cells. (E) Pretreatment with anti-JC blocking antibody does not inhibit PKH67 labeled EV internalization. (F) Titration of the anti-JCPyV antisera used to inhibit virus infection in panels D and E. * = p < 0.05.

Human macroglial cells (oligodendrocytes and astrocytes) in vivo do not express the sialic acid attachment receptor for JCPyV but cells growing in tissue culture including SVG-A and primary astrocytes express sialic acid receptors and bind virus. Because of this we used receptor destroying enzyme (type II neuraminidase) to remove the sialic acid containing virus receptors from the SVG-A cells and then compared their susceptibility to infection with purified virus particles or to infection by EV associated virus. Infection of the SVG-A cells by purified JCPyV was sensitive to neuraminidase treatment but transmission via EV was not **(Fig 5A)**. We also measured the uptake of labeled vesicles following treatment with neuraminidase. For these experiments the EV were labeled with PKH67 and incubated with untreated or neuraminidase treated cells before measuring trypan blue resistant uptake by flow cytometry **(S1 Fig)**. Neuraminidase had no significant effect on the uptake of EV isolated from CPEL cells into untreated cells consistent with the infectivity data **(Fig 5B).** As a control for the effectiveness of the neuraminidase we treated SVG-A cells with increasing concentrations of neuraminidase and measured the binding of purified and labeled JCPyV to the cells **(Fig 5C).** Neuraminidase reduced virus binding in a dose dependent manner **(Fig 5C).**

### EV-mediated infection of glial cells is not blocked by neutralizing antibodies

Incubation of the EV with neutralizing antibodies against JCPyV failed to inhibit EV mediated transmission but could inhibit infection by purified virus **(Fig 5D)**. We also measured the uptake of labeled vesicles following incubation with neutralizing antibody. Incubation of PKH67 labeled EV with anti-JCPyV antisera or with pre-immune antibody (PI) had no effect on EV uptake **(Fig 5E)**. As a control for the effectiveness of the antisera we pre-incubated purified virus with a dilution series of either preimmune control sera or anti-JCPyV sera. The antisera inhibits infection in a dose dependent manner **(Fig 5F).**

### EV mediated infection of SVG-A cells with externally associated virus can be blocked with anti-JCPyV antisera

**Fig 6:**
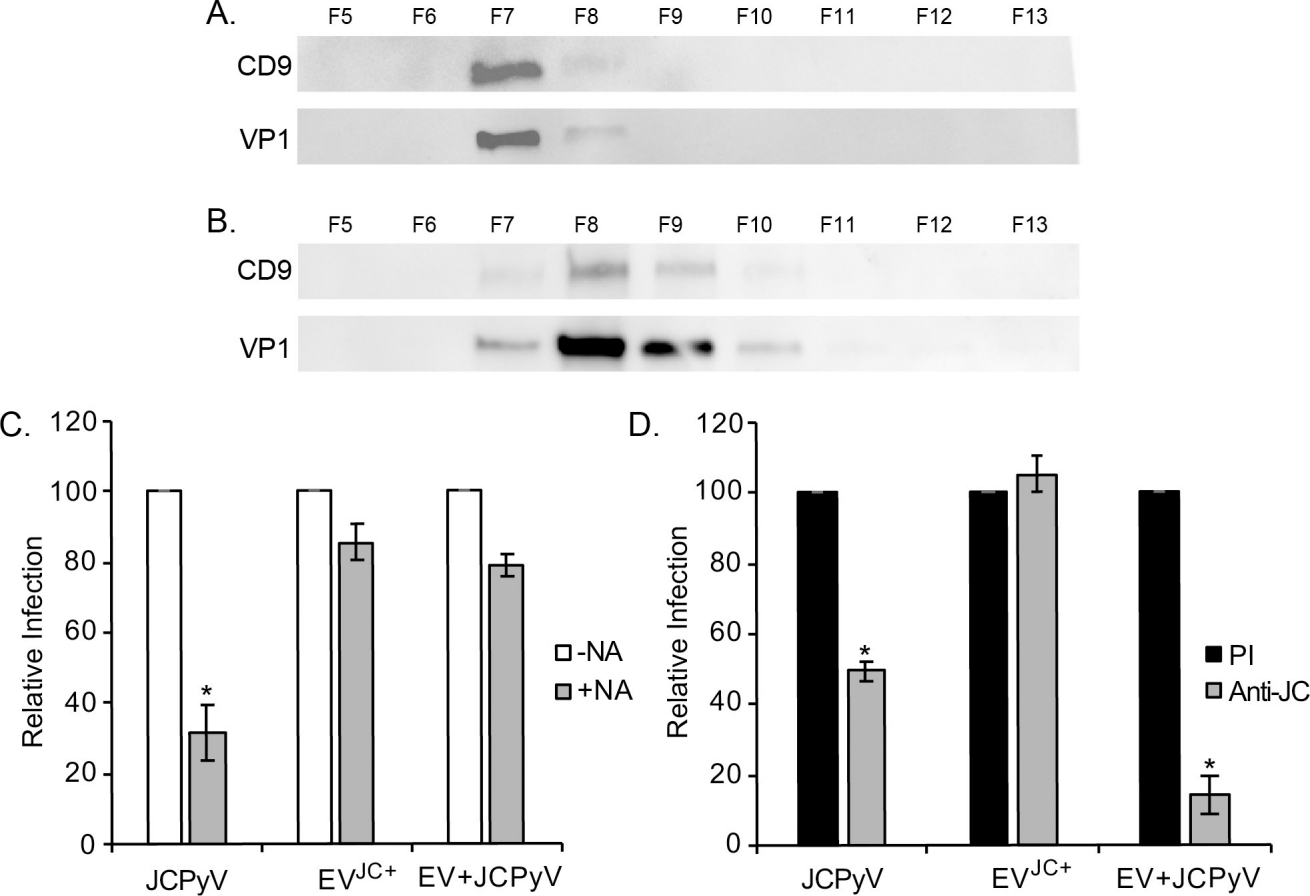
EV mediated infection of SVG-A cells with externally associated virus can be blocked with anti-JCPyV antisera. (A) EV^JC+^ from infected CPEL cells were concentrated by differential ultracentrifugation and purified over a qEV column. Western blot analysis of each fraction showed that the viral protein VP1 and the EV marker CD9 co-purifed in fraction 7. (B) EVs from uninfected CPEL cells were concentrated by differential ultracentrifugation and then spiked with 10e9 copies of purified JCPyV particles. This mixture was then subjected to fractionation over the qEV column and analyzed by western blot for CD9 and VP1. VP1 and CD9 co-purifed in fractions 7 and 8. (C) Fraction 7 from each was used to infect neuraminidase treated SVG-A cells. Neuraminidase only inhibited infection by purified JCPyV and had no effect on the transmission of virus by EV isolated from infected cells or from EV isolated from uninfected cells and spiked with purified virus. (D) Anti-JCPyV antisera (Anti-JC) but not preimmune sera (PI) inhibited infection by purified JCPyV and by EV isolated from uninfected cells and subsequently spiked with purified virus. Anti-JC had no effect on the transmission of virus from EV isolated from infected CPEL cells (EV^JC+^). * = p < 0.01.

Because we observed virus both inside of extracellular vesicles and attached to the outside of EV we asked whether EV with virus attached only to the outside behaved differently than EV with virus enclosed within the vesicles. To do this we purified extracellular vesicles from infected CPEL cells by differential ultracentrifugation (UC) followed by size exclusion chromatography (SEC). We also spiked UC purified EV isolated from uninfected CPEL cells with 10e9 copies of purified JCPyV particles and this mixture was then purified further by SEC. Fractions were collected and analyzed by Western blot for the vesicle marker CD9 and for the viral protein VP1. The virus co-purified with extracellular vesicle fractions in both cases **(Fig 6A and 6B, and S2 Fig)**. We then used these fractions to challenge uninfected SVG-A cells that had been treated with neuraminidase. Neuraminidase treatment of SVG-A cells reduced infection by purified JCPyV virions but had no significant effect on the transmission of virus from EVs isolated from infected cells (EV^**JC+**^) or by EVs from uninfected cells that were spiked with purified virus (EV + JCPyV) **(Fig 6C).** This indicates that the extracellular vesicle and not virus bound to the outside of the vesicle is driving infectious entry. We then asked whether virus bound only to the outside of the vesicles (EV + JCPyV) could be inhibited by neutralizing antisera. Neutralizing antibody was capable of inhibiting infection by EV containing virus bound only to the outside of the vesicle **(Fig 6D).** This is in contrast to EV derived from infected cells where virus inside of the extracellular vesicles are not sensitive to antibody neutralization (EV^**JC+**^) **(Fig 6C).**

### EV isolated from infected CPE cells enter by both clathrin dependent and independent mechanisms

**Fig 7:**
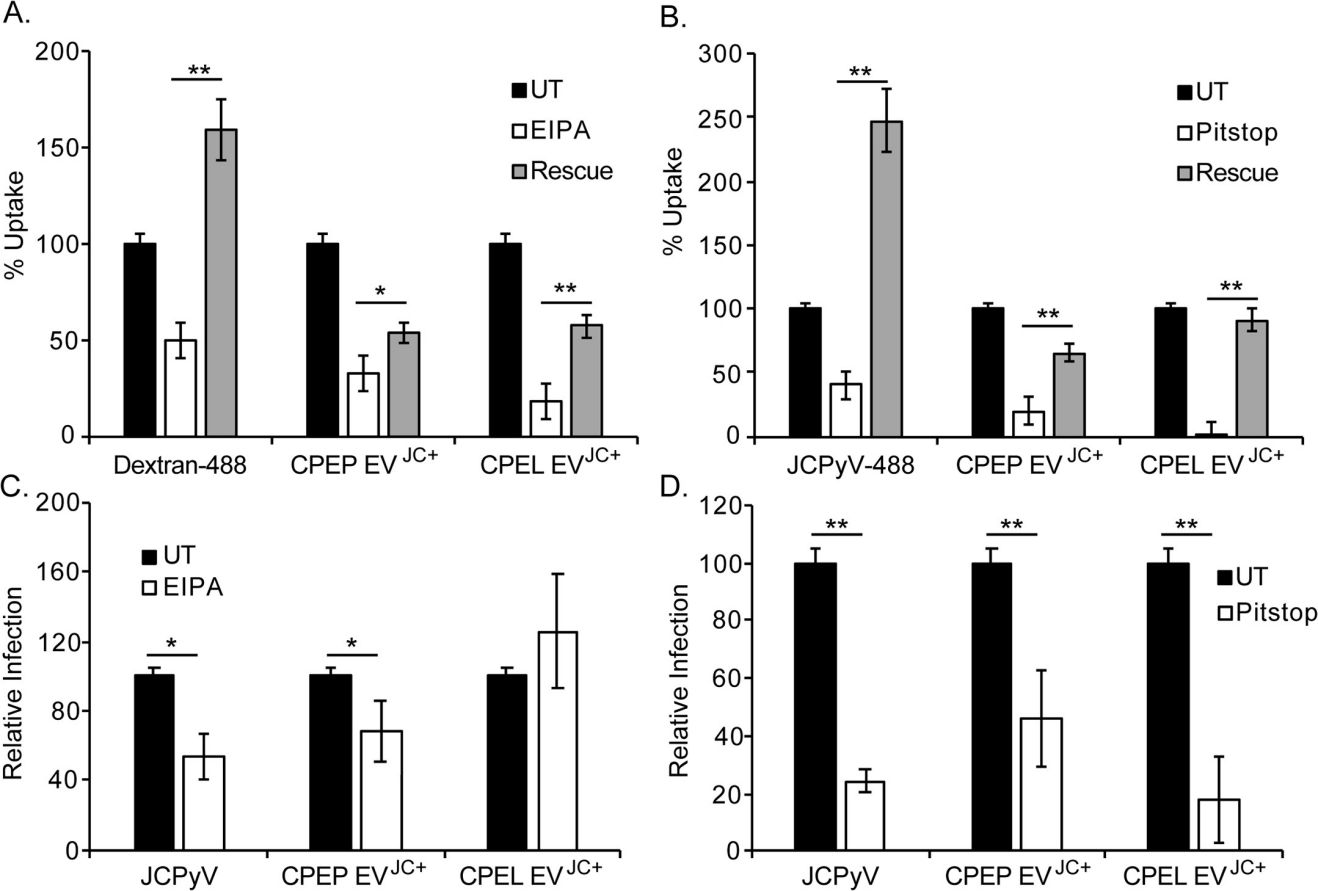
Infectious entry of EV isolated from infected CPE cells occurs by both clathrin dependent and independent mechanisms. (A) Pretreatment of SVG-A cells with the macropinocytosis inhibitor, EIPA. EIPA targets sodium/proton exchangers and effectively inhibits the uptake of labeled dextran (control) and labeled extracellular vesicles isolated from virus infected CPEP (CPEP EV^JC+^) and CPEL (CPEL EV^JC+^) cells into SVG-A cells. The inhibition of uptake is partially reversible by removing the drug and allowing the cells time to recover (Rescue) (B) Pretreatment of SVG-A cells with the clathrin mediated endocytosis inhibitor Pitstop2 effectively inhibits uptake of both purified JCPyV virus (control) and labeled extracellular vesicles isolated from virus infected CPEP (CPEP EV^JC+^) and CPEL (CPEL EV^JC+^) cells into SVG-A cells. The inhibition of uptake is partially reversible by removing the drug and allowing the cells time to recover (Rescue) (C) EIPA treatment of SVG-A cells reduces infection by purified virus and by infectious EV isolated from CPEP cells but not from CPEL cells. (D) Pitstop treatment of SVG-A cells reduces infection by purified virus and by infectious EV isolated from CPEP and CPEL cells. * p < 0.05; ** p < 0.01.

To determine how the extracellular vesicles deliver the virus across the plasma membrane of the target cells, EV from infected CPEP and CPEL cells were purified by UC and labeled with PKH67. These EV were then incubated with untreated SVG-A cells or with SVG-A cells that had been pre-treated with inhibitors of clathrin dependent endocytosis (Pitstop2), or macropinocytosis (EIPA). EV uptake was significantly reduced by the macropinocytosis inhibitor EIPA **(Fig 7A)** and by the clathrin dependent endocytic inhibitor Pitstop2 **(Fig 7B).** Alexa-fluor 488 labeled dextran was used as a control for the inhibition of macropinocytosis by EIPA and Alexa-fluor 488 labeled and purified JCPyV was used as a control for the clathrin dependent inhibitor Pitstop2. The controls for each pathway were inhibited by their respective drugs. Because the effects of EIPA and Pitstop2 are reversible we repeated the experiment and found that the inhibition of extracellular vesicle uptake into SVG-A cells was partially reversible when the drugs were removed and the cells allowed to recover for 2 hours before EV uptake **(Fig 7A and 7B)**. This indicates that the effect of the drugs is not due to toxicity or to off-target effects. The toxicity of each drug was also measured using an MTS assay **(S3 Fig)**. To determine whether a reduction in EV uptake correlated with a reduction in EV mediated infection we measured EV mediated infection at 4 days post-exposure to EV. We found that pre-treatment with EIPA modestly reduced infection with EV derived from CPEP cells but had no effect on infection of EV derived from CPEL cells **(Fig 7C).** In contrast, Pitstop significantly inhibited infection by EV derived from both cell types **(Fig 7D).** Purifed JCPyV was used as a positive control in these experiments and as expected was less inhibited by EIPA than by Pitstop **(Fig 7C and 7D).**

## Discussion

The choroid plexus sits at the interface between the blood and the cerebral spinal fluid (CSF) and is perfectly situated to play a role in delivering virus to the CNS in extracellular vesicles [31]. The choroid plexus is also known to play an important role in modulating immune responses in the CNS so it is reasonable to suspect that it could play a similar role in restricting viral growth under normal circumstances [25, 31]. These functions may be partially disrupted in patients who are profoundly immunosuppressed or who are treated with immunomodulatory drugs. Viruses such as JCPyV likely take advantage of these conditions to facilitate trafficking to brain parenchyma.

**Fig 8:**
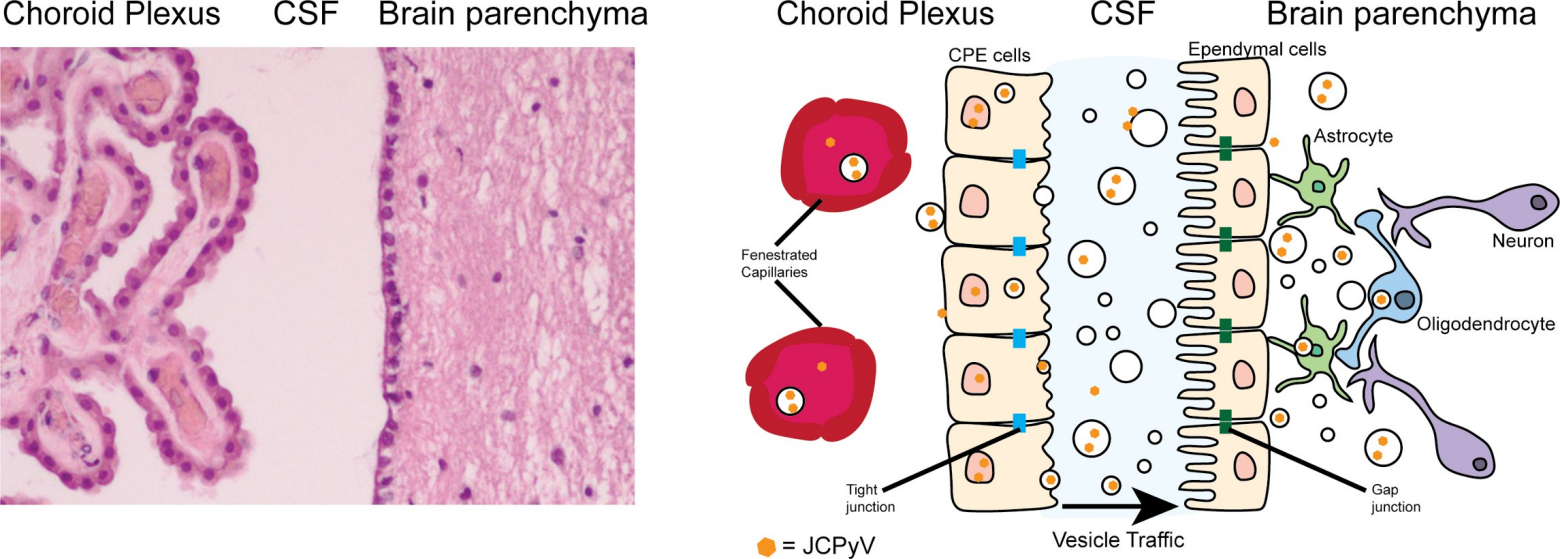
Model of JCPyV trafficking in EV to the CNS via the choroid plexus. Left panel: Hematoxylin and Eosin [H&E] stain of normal human choroid plexus and brain parenchyma; magnification: 200x. Right panel: Proposed model of JCPyV trafficking to the brain via the choroid plexus. JCPyV (either free virions or enclosed in vesicles) enters the stroma from the bloodstream through fenestrated capillaries to infect the choroid plexus epithelium. Infected CPE packages JCPyV into vesicles and secretes these EVs into the CSF, which subsequently enter the brain parenchyma and infect target glial cells.

Our central hypothesis is that the choroid plexus is important in the pathophysiology of JCPyV induced CNS disease by being a primary target of viral infection and by playing an important role in extracellular vesicle mediated spread of virus to brain parenchyma **(see the model, Fig 8)**.

Our hypothesis is supported by the in vivo demonstration of JCPyV infected choroid plexus epithelial cells and meningeal cells from a patient with meningitis and from several patients with PML [28, 29]. We also previously reported that the choroid plexus, like cells in the kidney, express the JCPyV attachment and entry receptors, bind virus in human tissue sections, and are completely susceptible to virus infection in vitro [17, 27]. This is in contrast to human macroglial cells that do not express the critical attachment receptor for the virus and do not bind virus in human tissue sections [17]. Other groups have shown that amino acids in the major virus capsid protein, VP1, known to be important for binding to the attachment receptor are often mutated in virus genomes isolated from the CSF and brain of patients with disease [18–20]. These data together with our finding that macroglia, the major targets of the virus in the CNS, do not express the attachment receptor indicates that there is no selective pressure to maintain sialic binding once the virus is in the CNS.

Because of the limited lifespan of primary cells in culture we immortalized primary choroid plexus epithelial cells with human telomerase (hTert) and showed that they maintain the properties of the primary cells. The immortalized cells were equally susceptible to JCPyV infection and expressed the choroid plexus marker transthyretin. We next isolated extracellular vesicles from both the primary CPE cells and the CPE cell line. The EV were produced in similar quantity and had a similar size distribution consistent with them being characterized as small extracellular vesicles. They also both expressed extracellular vesicle markers and were devoid of markers of contaminating small cellular organelles. TEM analysis of the extracellular vesicles isolated from virus infected CPE cells showed a heterogeneous population of EV with some having virus enclosed within the EV and others having virus attached only to the outside of the EV. This mixed population of EV readily transmitted the infection to human glial cells and the infection could not be blocked by treating the target cells with receptor destroying enzyme (Type II Neuraminidase). This indicates that vesicle mediated infection is independent of the known virus attachment receptor and is consistent with previous work showing that vesicles can transmit sialic acid mutant pseudoviruses to human glial cells [32]. Also, EV-mediated transmission of the infection was not neutralized by anti-viral antisera suggesting that this may be a mechanism for the virus to escape humoral immune responses. Serological studies show that the antibody response is not sufficient to prevent the development of PML as increases in antibody titer are not protective [33]. Virus containing mutations in and around the sialic acid binding pocket have also been shown to not be recognized by either the humoral or cell-mediated immune response and groups have postulated that this is another mechanism of immune escape [34, 35]. Because we found virus both within vesicles and attached to the outside we wanted to determine whether the virus on the outside of the vesicle played any role in infectious entry or whether infection was regulated by the vesicle to which the virus was attached. Because we could not physically separate these vesicles from the population we set up an experiment where vesicles from uninfected CPE cells were spiked with purified virus giving us a pure population of vesicles with virus only attached to the outside. These mixtures as well as vesicles derived from virus infected CPE cells were purified by size exclusion chromatography and fractions that were positive for the vesicle marker CD9 and for the viral protein VP1 were used to challenge uninfected glial cells treated with or without neuraminidase. Neuraminidase had no effect on the ability of either preparation to transmit the infection to glia whereas infection with purified virus was significantly reduced. This indicates that it is the vesicle driving infectious entry not the virus particle bound to the outside. One clear difference we did observe was that infection of cells by EV containing only virus bound to the outside were significantly inhibited by anti-JCPyV antisera indicating that virus remained exposed to the antibody.

We also investigated the mechanisms responsible for uptake of EV containing virus into target cells. The uptake assay was done by incubating untreated and treated cells with PKH67 labeled vesicles containing virus and adding trypan blue to each sample immediately prior to flow cytometric analysis. This allowed us to score the fluorescent signal from labeled EV that successfully internalized into the cell and escaped the trypan blue quench. Any labeled EV remaining at the surface or any labeled EV that directly fused with the plasma membrane would be quenched by trypan blue in this assay. An inhibitor of macropinocytosis (EIPA) and an inhibitor of clathrin dependent endocytosis (Pitstop2) reduced vesicle uptake and vesicle mediated infection into target cells. The effect of the drugs was partially reversible when drugs were removed and cells allowed to recover for two hours before the addition of the labeled EV. It is possible that there are two or more distinct populations of EV that enter cells by different mechanisms or perhaps virus is associated with some but not all vesicles that enter cells by one or both pathways. Interestingly, van der Grein recently demonstrated that picornaviruses are associated with multiple subsets of extracellular vesicles that differ in molecular composition and in infectivity [36].

Other non-enveloped viruses, including picornaviruses and noroviruses have recently been shown to take advantage of extracellular vesicles to promote their transmission [36–42]. This mode of infection has been shown to be more efficient than infection by individual viral particles due to the increased multiplicity of infection of a vesicle containing many virions vs single virions in isolation [42]. Our observations are similar as vesicles derived from infected CPE cells are much more infectious than purified virus particles. Moreover, the virus enclosed within the extracellular vesicles are protected from humoral immune responses and are capable of transmitting the infection to cells that lack the virus attachment receptor and would otherwise not be susceptible to infection.

Because CPE-derived vesicles are known to play a role in blood-brain communication we suspect that this is a major mechanism for JCPyV delivery from the periphery to the brain to cause disease **(Fig 8)**. Once in the brain vesicles likely play a fundamental role in transmitting the infection to other glial cells and perhaps also to neurons in the case of granule cell neuronopathy, a rare complication of JCPyV infection of the brain [43]. Extracellular vesicles are also known to carry an abundance of regulatory molecules including transcription factors and microRNAs that influence the microenvironment of target tissues [44–50]. The contents of EV derived from JCPyV infected CPE cells, kidney tubule epithelial cells, and primary glial cells has yet to be examined in vitro or in vivo but such studies are likely to shed significant light on the pathogenic mechanisms of JCPyV induced disease. The identification of a molecular signature on choroid plexus and glial cell extracellular vesicles associated with disease could lead to the development of biomarkers that are predictive of disease initiation or progression as has been shown in other infectious and non-infectious diseases of the CNS [51–53].

## Materials and methods

### Cells and virus propagation

SVG-A cells (SV40 T antigen transformed glial cells-astrocyte) are a subclone of the human glial cell line SVG (SV40 T antigen transformed glial cells) transformed with an origin-defective SV40 mutant originally obtained from Dr. E.O. Major [54]. SVG-A cells were grown in Minimum Essential Medium supplemented with 10% fetal bovine serum (FBS) and 1% antibiotic/antimycotic (Mediatech). HEK293T (Human embryonic kidney T antigen transformed) were obtained from ATCC (catalog #CRL-11268: RRID:CVCL_1926) and were grown in Dulbecco’s minimal essential medium (DMEM) (Corning) supplemented with 10% fetal bovine serum (FBS), 1% nonessential amino acids (Gibco Life Technologies), and 1% antibiotic/antimycotic. Choroid plexus epithelial cells (ScienCell Research Labs, catalog #1310) and primary human astrocytes (ScienCell Research Labs, catalog #1800) were maintained in the supplier-recommended media as appropriate for each cell type (ScienCell Research Labs). Cells were grown in a humidified chamber at 37°C and 5% CO_2_.

Generation, propagation and purification of the Mad-1/SVE strain of JCPyV were performed as previously described [55–57]. Alexa Fluor 488-labeled carboxylic acid-succinimidyl ester (ThermoFisher/Invitrogen) was used to label purified JCPyV. Briefly, 5.0 μg of cesium chloride purified virus was dialyzed against labeling buffer (0.1 M NaHCO_3_; pH 8.3) at 4°C overnight in 10,000 MWCO cartridges (Pierce). The virus was then incubated for 1 hour with 0.5 μg of Alexa Fluor 488-labeled succinimidyl ester (AF488) in 100 μl of dimethyl sulfoxide (DMSO). The AF488-labeled virus was extensively dialyzed in 10,000 MWCO cartridges against two changes of buffer A (10 mM Tris-HCl, 50 mM NaCl, 0.1 mM CaCl_2_) at 4°C for an additional 48 hours to remove excess dye.

EV-depleted medium (EV-D) was used as needed for vesicle related experiments and vesicle production, while complete medium was used for general passage of cell lines. EV-depleted medium was prepared at 2x and spun at 100,000×*g* in a type 45 Ti rotor (*k* factor = 133) for 18 hours. Medium was then diluted before use to 1x and filtered through a 0.22 μm pore filter (Celltreat, Pepperell, MA) [58].

Plasmids pLV-hTERT IRES hygro (85140), pCMVdr8.9 (8455), and pVSV-G (8454) were purchased from Addgene. 5-(*N*-Ethyl-*N*-isopropyl) amiloride (A3085, 100 μM), Neuraminidase Type II (N6514, 0.5 U/ml) and PKH67 labeling kit (MIDI67) were purchased from Sigma. Pitstop2 was purchased from Abcam (ab120687, 25 μM). Hygromycin B was purchased from Thermo/Invitrogen (10687010, 25 μg/ml).

### hTert immortalization

Primary human choroid plexus cells were immortalized as previously described [59]. Early passage primary CPE cells were serially infected with hTert lentiviral particles once per day for 3 consecutive days. Following infection, cells were recovered in EpiCM media for 48 hours, followed by selection in EpiCM with 25μg/ml hygromycin for an additional 7 days. Following selection, primary CPE cells and hTert immortalized CPE cells were authenticated using the ATCC Human STR Profiling Cell Authentication service.

### Purification of extracellular vesicles

EVs were concentrated by differential centrifugation of CPE cell supernatant [60]. Debris was pelleted at 300×*g* in a Sorvall Legend X1R (Thermo) centrifuge for 10 minutes, followed by a 2,000 × *g* spin for 10 minutes. Supernatant was spun twice at 10,000 × *g* in a Sorvall Lynx 6000 (Thermo) centrifuge for 30 minutes each. Supernatant was then transferred to Ultra Clear tubes (Beckman Coulter, Brea, CA) and spun at 100,000 × *g* for 70 minutes in a SW55 Ti rotor (*k*-factor = 139) or 2 hours in a SW41 Ti rotor (*k*-factor = 256). The pellet was washed with phosphate-buffered saline (PBS) and centrifuged again at 100,000 × *g*. The pellet was suspended in sterile PBS at 1/200^th^ of original volume and stored at 4°C for short-term storage (5 days or less) or at −20°C for longer term storage. All centrifugation steps were carried out at 4°C. Supernatant was transferred to a clean tube after each centrifugation step using a pipet. EV preparations were characterized by Western blotting, TEM, and nanoparticle tracking analysis (NTA).

For purification by size exclusion chromatography (SEC), qEV original columns (IZON, 500 μl sample max, 35 nm cutoff) were equilibrated to room temperature and washed with three column volumes (10 ml) of 0.22 μM filtered PBS. 250 μl vesicles from naïve cells spiked with purified virus or from infected CPE cells were loaded directly onto the sample frit and allowed to enter the column, per the manufacturer’s instructions. Fractions were collected in 500 μl volumes, starting with the void volume, for a total of 15 fractions per sample. Fractions were analyzed by NTA, Western blot, and protein concentration (Qubit) and used to infect SVG-A cells.

### Nanoparticle tracking analysis

Nanoparticle tracking analysis (NTA) was performed using a Malvern NanoSight NS300 instrument. Samples and standards were diluted in 0.1 μM filtered and degassed PBS. Nanosphere size standards (125nM, Thermo) were diluted to 1:5,000 and 1:10,000 and used to perform initial focus for the NTA reading, followed by washing with degassed PBS. After each sample was processed, the lines were thoroughly washed again with PBS. Parameters were set as follows: flow rate of 50 μl/min, with five 30-s videos recorded for analysis.

### Transmission electron microscopy

Transmission electron microscopy (TEM) was performed as described previously [38], with modifications. Briefly, 5 μl of sample was adsorbed to a Formvar carbon-coated 400-mesh copper grid for 15 minutes. The grid was then inverted onto a drop of 1% glutaraldehyde for 1 minute and then transferred to fresh drops of distilled water 3 times for 2 minutes each time. The grid was inverted onto a drop of 3% ammonium molybdate for 2 minutes to enable the contrast process. The sample was wicked, allowed to dry, and imaged at 100 kV on a Philips 410 transmission electron microscope.

### Virus infection

Cells were plated at 10,000 cells/cm^2^ and infected in serum-free media for 1 hour at 37°C. After infection, inoculum was aspirated and replaced with complete, EV-depleted media. For initial characterization, CsCl purified JCPyV was used to infect SVG-A and CPE cultures for 1 hour at 37°C. To generate EVs, supernatants were collected from either infected or uninfected CPEP and CPEL cells at 7 days post-infection. SVG-A, primary human astrocytes and HEK293T cells were challenged with virus or EV^JC+^ for 1 hour at 37°C. For infection inhibition experiments, SVG-A cultures were pretreated with EIPA or Pitstop for 1 hour at 37°C. CsCl purified JCPyV and EV^JC+^ were used to infect SVG-A cultures in the continued presence of drug. After infection, inoculum was aspirated and replaced with complete, EV-depleted media containing EIPA or Pitstop. Cells were shifted to 37°C and stained for VP1 at 4 days post infection.

For the neuraminidase experiments, Type II neuraminidase (Sigma) was used at 0.5 U/ml to treat cells for 1 hour at 37°C at pH 6.0. For the antiserum experiment, virus or EV^JC+^ were pretreated with anti-JCPyV blocking antibody or pre-immune serum at 1:10,000 for 1 hour at 4°C. For titrating antiserum, purified virus was pretreated with blocking antibody or pre-immune serum at 1:1,000, 1:10,000 and 1:50,000 dilutions. Following pretreatments, SVG-A cells were prechilled at 4°C for 30 minutes, infected with antibody/virus or antibody/ EV^JC+^ complexes on ice, and allowed to bind for an additional hour. After infection, cells were washed with PBS and incubated in complete, EV-depleted media containing anti-JCPyV blocking antibody or pre-immune control diluted to 1:10,000. Cells were shifted to 37°C and stained for VP1 at 4 days post infection.

### Indirect immunofluorescent analysis

To score infection by indirect immunofluorescent, cells were washed in PBS, fixed in 100% ice cold methanol (MeOH), and incubated at −20°C for 30 minutes. Fixed cells were washed in PBS and allowed to rehydrate for 15 minutes, and incubated with VP1-specific antibody PAB597 (1:50) in PBS at 37°C for 1 hour. Following primary antibody incubation cells were again washed with PBS incubated with a goat anti-mouse AF488 (1:1,000) conjugated antibody in PBS at 37°C for 1 hour. Secondary antibody was washed out and cells were counterstained with DAPI (1:1000) in PBS for 10 minutes at room temperature. Cells were analyzed for nuclear VP1 staining and total cell number under a 20x objective using a Ti2-E fluorescent microscope (Nikon). Cell count analysis was performed using Elements High Content imaging software (Nikon).

For characterization, CPEP and CPEL cells were fixed for 30 minutes at room temperature in 4% paraformaldehyde (PFA). Following fixation, cells were permeabilized with 0.5% Triton X-100 (TX-100, USB Corporation) at room temperature for 10 minutes. Fixed samples were incubated overnight with transthyretin primary antibody (Dako, A0002) in PBS (1:250, overnight at 4°C) followed by PBS washing and incubation with goat anti-rabbbit AF488 (1:1,000) at 37°C for 1 hour. Samples were counterstained with DAPI and imaged using Elements High Content imaging software on a Ti2-E fluorescent microscope (Nikon). The percentage of VP1 positive cells was determined using the Nikon High Content Analysis software to count and calculate the ratio of VP1 positive cells compared to DAPI positive cells.

### Antibodies

PAB597 is a hybridoma supernatant that produces a monoclonal antibody against JCPyV VP1 [61]. AF488 labeled goat anti-mouse 488 or anti-rabbit antibodies (Thermo/Invitrogen) were used as the secondary antibodies for immunofluorescence experiments. Transthyretin primary antibody (Dako, A0002) was used for initial characterization of CPE cells (1:250, overnight at 4C) followed by AF488 labeled goat anti-rabbit antibody (1:1,000). Primary antibodies and the respective dilutions used for Western blotting included annexin V (Abcam, ab117439, 1:1,000), CD9 (Cell Signaling Technologies, CST 13174S, 1:1,000), flotillin-1 (CST 18634S, 1:1,000), GM130 (Cell Signaling Technologies, CST 12480, 1:1,000), calnexin (Santa Cruz Biotechnology, TX; sc-11397, 1:200), cytochrome *c* (BD Pharmingen 556433, 1:500), TSG101 (Thermo PA5-31260, 1:500), and PAB597 (1:2,000). Secondary antibodies used for Western blotting anti-mouse horseradish peroxidase (HRP) (Thermo A28177) and anti-rabbit HRP (Thermo A27036), both used at 1:10,000.

### Western blots

Samples were lysed on ice in Pierce radioimmunoprecipitation assay (RIPA) buffer (Thermo, 89900) containing complete EDTA-free protease inhibitor cocktail (Sigma/Roche, 11836170001) and debris was pelleted. Protein content was determined using Pierce Micro bicinchoninic acid (BCA) Protein Assay kit (Thermo, 23235). Samples were prepared in 4x loading dye (Bio-Rad Laboratories, Hercules, CA), boiled at 95°C for 5 min, and loaded in 4%-15% gradient Mini-Protean TGX Stain-Free precast gels (Bio-Rad). Gels were run at 175V and transferred to a 0.2-μm-pore-size nitrocellulose membrane by the semidry transfer method. Blots were blocked in 1% casein buffer/Tris-buffered saline (TBS) for 3 hours at 4°C. Primary antibodies were diluted in 1% casein and incubated overnight at 4°C. Blots were washed three times with TBS with 0.01% Tween 20 (TBST) and incubated with secondary antibody diluted in TBST for 1 hour at room temperature. Horseradish peroxidase secondary antibodies were diluted 1:10,000. Blots were washed three times with TBST and then incubated with ClarityMax according to manufacturer’s recommendation (Bio-Rad, 1075062) for HRP secondary antibodies. Blots were imaged on a ChemiDoc MP imaging system.

### Extracellular vesicle labeling and uptake

Purified EVs were labeled immediately prior to each experiment with PKH67 fluorescent labeling dye (Sigma), according to the manufacturer’s recommended protocol. SVG-A cells were plated to 24-well dishes at 100,000 cells/well in serum free EV-D media, as required per experiment. For the neuraminidase experiment and blocking antibody experiment pretreatments were conducted as described for infections. For uptake inhibitors, the following doses were used; Pitstop2 25μM; EIPA 100μM. Purified, AF488-labeled JCPyV or AF488 conjugated Dextran (Invitrogen) were used as controls. Drugs were incubated with SVG-A in serum free (Fig 7) media for 2 hours, after which labeled EVs were added (4.0e8 particles per dose) and incubated for an additional 2 hours at 37°C. Following incubation, media was discarded, cells were washed in PBS and harvested with 250μL trypsin. Cells were pelleted, washed again and suspended in PBS. Trypan blue was added (final concentration 0.016%) to each sample immediately prior to analysis. Trypan blue was utilized to exclude both non-specific entry into dead cells and/or incompletely internalized signal from labeled EVs or virus. Flow cytometry was performed using a BD FACSCanto II (BD Biosciences) and 10,000 events were collected for each sample.

For the rescue experiments, after a 2 hour pretreatment, drug media was replaced with EV-D media and cells were allowed to recover for 2 hours at 37°C with three changes of media. Following recovery, labeled EV, virus or dextran was added and allowed to internalize for 2 hours at 37°C. Cells were harvested with trypsin, extensively washed, suspended in PBS and analyzed with trypan blue as described above.

### Quantification of protected viral genomes by qPCR

Protected viral genome content of each sample was quantified by qPCR. EV^JC+^ were first treated with DNAse 1 (New England Biolabs) for 30 minutes at 37°C, followed by a 10 minute inactivation at 75°C according to the manufacturer’s protocol. Pretreatment with DNAse removes non-encapsidated and contaminating DNA. Samples were next processed using a Blood and Tissue Kit (Qiagen) to disrupt EV and capsid protein. Quantitative PCR was conducted using VP2 Taqman Assay primer/probe set (probe: /5HEX/TGTTCTCCA/ZEN/CAATCTCCCAGGCTT/3IABkFQ/ primer 1: CCTGGAGTGAATGCCTTTGT primer 2: AGAGGTTAAGGCTGGCAAATC) (IDT) and run on a BioRad CFX96 detection system. Genome number was calculated by comparison to a standard curve of JCPyV DNA.

### Flow cytometry and binding

SVG-A cells were plated to 24-well dishes at 100,000 cells/well. Cells were collected using Cellstripper (Corning/Cellgro, Inc.), washed extensively in PBS and treated with increasing concentrations of Type II neuraminidase (NA) for 1 hour at 37°C at pH 6.0. Following treatment, cells were washed using PBS and bound with AF488-labeled JCPyV for 1 hour on ice. Unbound virus was washed out with PBS. Flow cytometry was performed using a BD FACSCanto II (BD Biosciences) and 10,000 events were collected for each sample.

Data was analyzed using FlowJo and is expressed at the percentage of AF488-labeled JCPyV positive cells versus the untreated, JCPyV-488 bound control.

### Tissue labeling

Formalin fixed, paraffin embedded (FFPE) human brain sections including choroid plexus were deparaffinized in xylene followed by a series of graded ethanol washes and then stained with hematoxylin and eosin.

Images were captured by Spot RT digital imaging (Sterling Heights, Michigan) on a Nikon E600 microscope (Melville, NY) with brightfield optics.

#### Statistical Analysis

Means for triplicate samples were compared using an unpaired Student’s *t* test. Error bars represent the standard deviation from 3 independent experiments. *P* values <0.05 were considered statistically significant.

### Ethics statement

Anonymized human brain and choroid plexus samples were obtained from Rhode Island Hospital in accordance with protocols approved by the Institutional Review Boards at Brown University and Rhode Island Hospital. IRB approval #240615–1.

## Supporting information

S1 Table STR analysis of CPEP and CPEL cells. CPEP and CPEL cells were authenticated using Short Tandem Repeat (STR) analysis as described in 2012 in ANSI Standard (ASN-0002) Authentication of Human Cell Lines: Standardization of STR Profiling by the ATCC Standards Development Organization. Seventeen short tandem repeat (STR) loci plus the gender determining locus, Amelogenin, were amplified using the commercially available PowerPlex 18D Kit from Promega. Samples were processed using the ABI Prism 3500xl Genetic Analyzer. Data were analyzed using GeneMapper ID-X v1.2 software (Applied Biosystems). The CPE line shares 100% similarity with the parental CPE primary cells.
(TIF)

S1 Fig Trypan blue quenching assay. PKH67 labeled extracellular vesicles were allowed to bind to CPE cells at 4°C. The addition of trypan blue (+ TB) completely quenched the signal as seen in the fluorescent micrographs and in the histograms obtained by flow cytometric analysis. When cells were shifted to 37°C EV are internalized and the addition of trypan blue has no effect on the intracellular signal.
(TIF)

S2 Fig Infectivity of SEC fractions. **(A)** Extracellular vesicles from JCPyV infected CPEL cells were purified by ultracentrifugation and size exclusion chromatography (SEC). SEC fraction 5–13 were used to challenge SVG-A cells. Infectivity was scored by indirect immunofluorescence analysis of VP1 positive cells (green). The cells were counterstained with DAPI. Fractions 7 and 8 contained the majority of infectious extracellular vesicles. (B) Extracellular vesicles from uninfected CPEL cells were purified by ultracentrifugation and then spiked with purified JCPyV virion particles. This mixture was then further purified by SEC and the resulting fractions tested for infectivity. Fractions 8 and 9 contained the majority of infectious extracellular vesicles but infectious material also was apparent in fractions 10–13.
(TIF)

S3 Fig MTS assay of Pitstop2, and EIPA treated SVG-A cells. An MTS assay was used to assess the metabolic activity of cells being treated with compounds that antagonize specific cellular entry pathways. None of the compounds used negatively affected metabolic activity of the cells at the concentrations used in the uptake assays.
(TIF)
