# Supplementary figures and images for: JC Virus infected choroid plexus epithelial cells produce extracellular vesicles that infect glial cells independently of the virus attachment receptor
Source: PLoS Pathog. 2020 Mar 4;16(3):e1008371. doi: 10.1371/journal.ppat.1008371 (PMC7075641; doi:10.1371/journal.ppat.1008371)

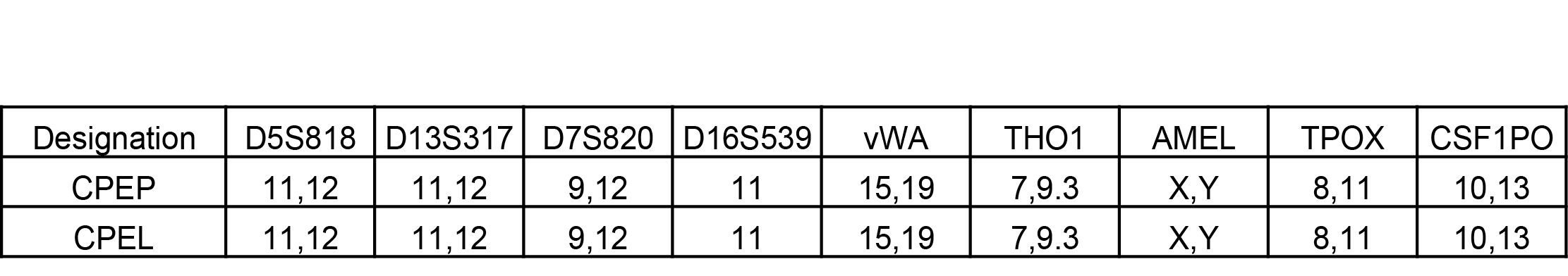

Supplement: S1 Table — CPEP and CPEL cells were authenticated using Short Tandem Repeat (STR) analysis as described in 2012 in ANSI Standard (ASN-0002) Authentication of Human Cell Lines: Standardization of STR Profiling by the ATCC Standards Development Organization. Seventeen short tandem repeat (STR) loci plus the gender determining locus, Amelogenin, were amplified using the commercially available PowerPlex 18D Kit from Promega. Samples were processed using the ABI Prism 3500xl Genetic Analyzer. Data were analyzed using GeneMapper ID-X v1.2 software (Applied Biosystems). The CPE line shares 100% similarity with the parental CPE primary cells. (TIF) [file ppat.1008371.s001.tif]

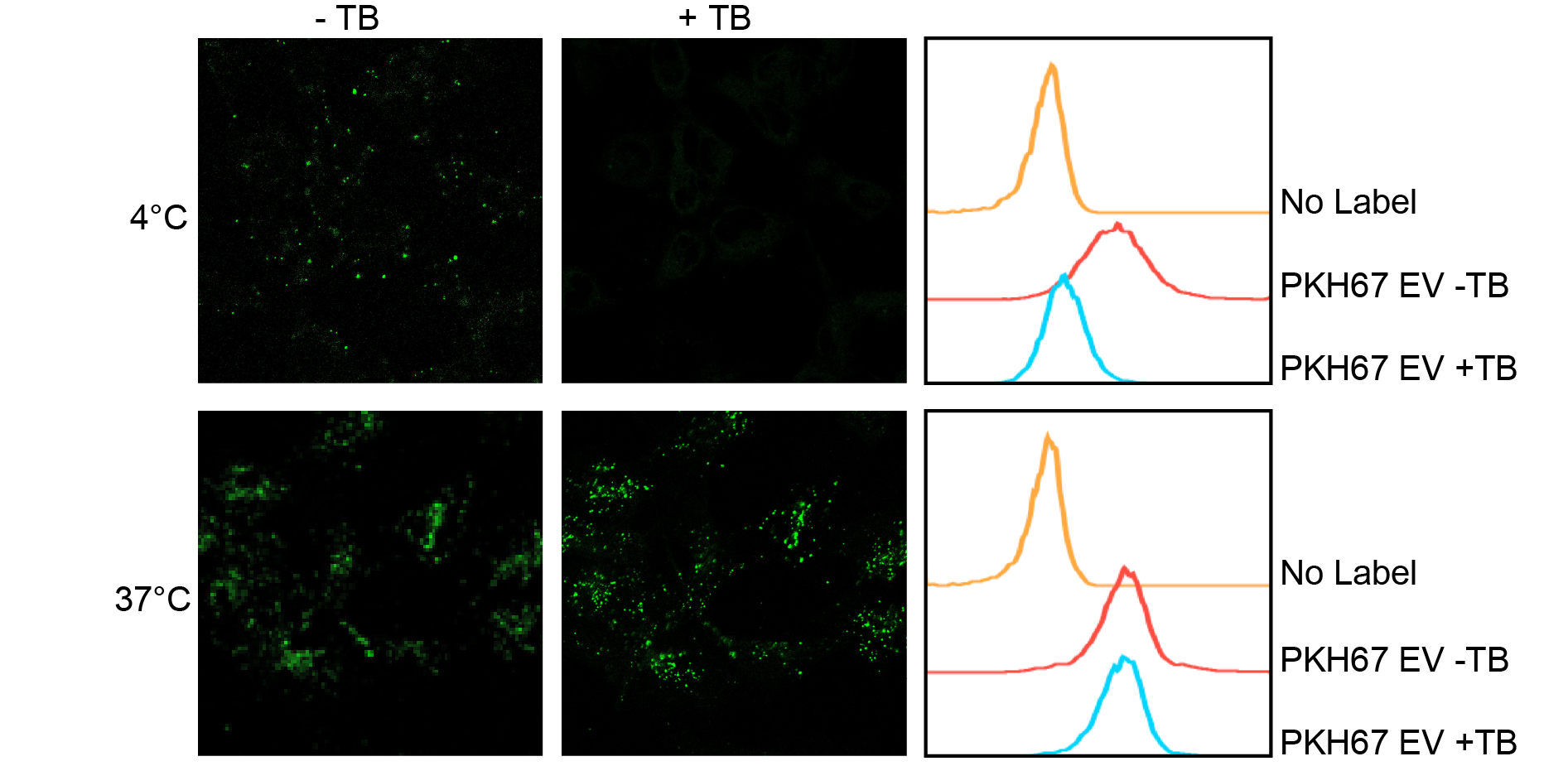

Supplement: S1 Fig — PKH67 labeled extracellular vesicles were allowed to bind to CPE cells at 4°C. The addition of trypan blue (+ TB) completely quenched the signal as seen in the fluorescent micrographs and in the histograms obtained by flow cytometric analysis. When cells were shifted to 37°C EV are internalized and the addition of trypan blue has no effect on the intracellular signal. (TIF) [file ppat.1008371.s002.tif]

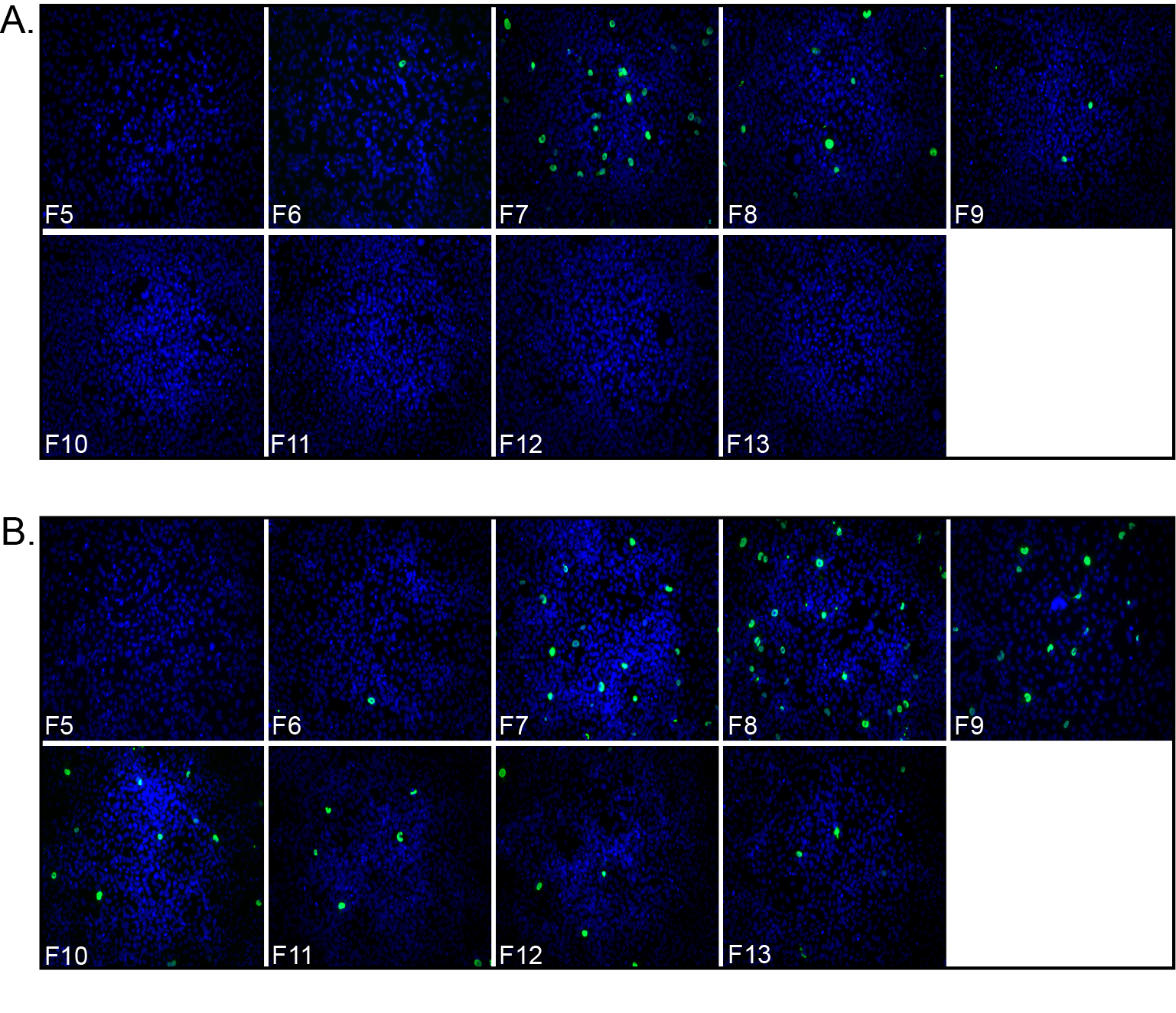

Supplement: S2 Fig — (A) Extracellular vesicles from JCPyV infected CPEL cells were purified by ultracentrifugation and size exclusion chromatography (SEC). SEC fraction 5–13 were used to challenge SVG-A cells. Infectivity was scored by indirect immunofluorescence analysis of VP1 positive cells (green). The cells were counterstained with DAPI. Fractions 7 and 8 contained the majority of infectious extracellular vesicles. (B) Extracellular vesicles from uninfected CPEL cells were purified by ultracentrifugation and then spiked with purified JCPyV virion particles. This mixture was then further purified by SEC and the resulting fractions tested for infectivity. Fractions 8 and 9 contained the majority of infectious extracellular vesicles but infectious material also was apparent in fractions 10–13. (TIF) [file ppat.1008371.s003.tif]

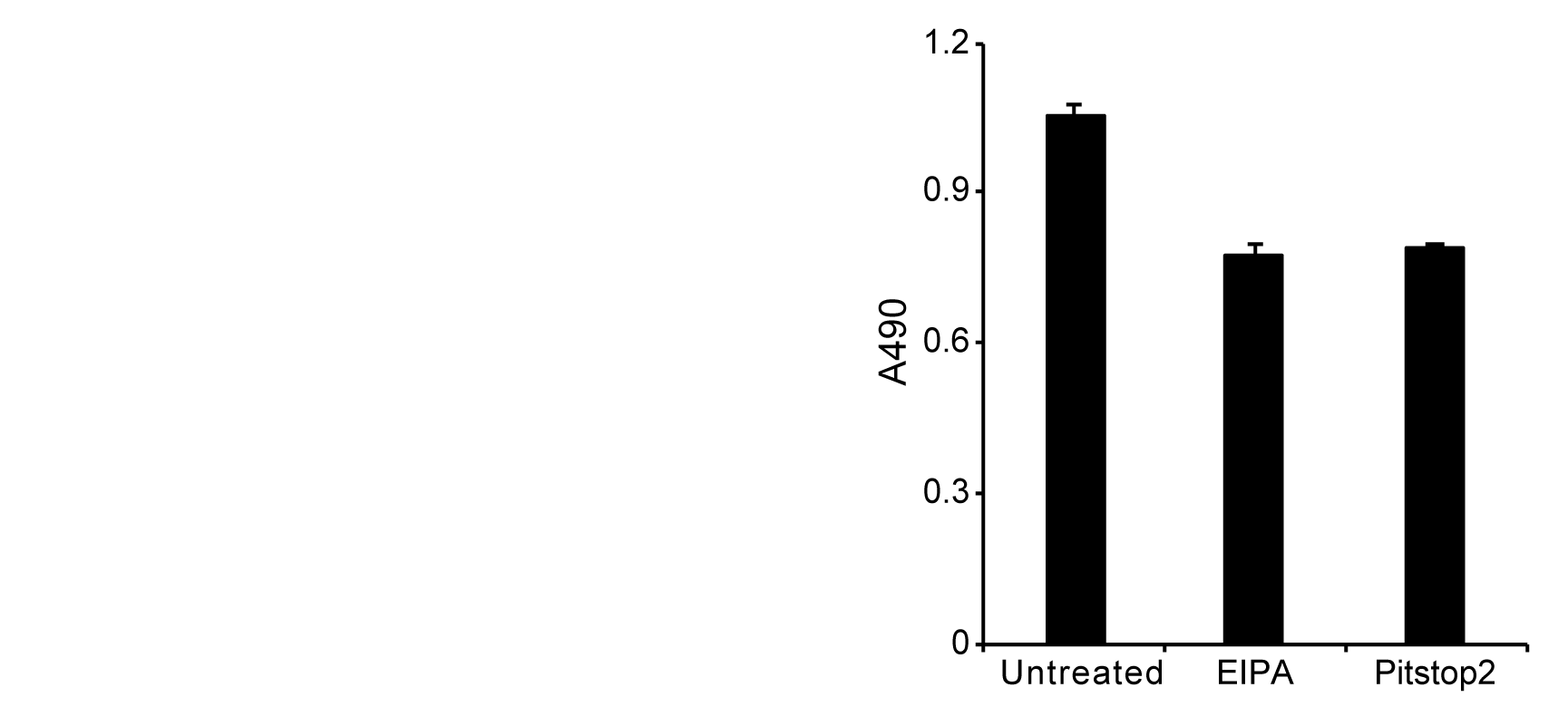

Supplement: S3 Fig — An MTS assay was used to assess the metabolic activity of cells being treated with compounds that antagonize specific cellular entry pathways. None of the compounds used negatively affected metabolic activity of the cells at the concentrations used in the uptake assays. (TIF) [file ppat.1008371.s004.tif]
